# Multiplex networks of cortical and hippocampal neurons revealed at different timescales

**DOI:** 10.1186/1471-2202-15-S1-P212

**Published:** 2014-07-21

**Authors:** Nicholas Timme, Shinya Ito, Maxym Myroshnychenko, Fang-Chin Yeh, Emma Hiolski, Alan M Litke, John M Beggs

**Affiliations:** 1 Department of Physics, Indiana University, Bloomington, IN 47405, USA; 2 Santa Cruz Institute for Particle Physics, University of California at Santa Cruz, Santa Cruz, CA 95064 USA; 3 Microbiology & Environmental Toxicology Department, University of California at Santa Cruz, Santa Cruz, CA 95064 USA

## 

**Figure 1:**
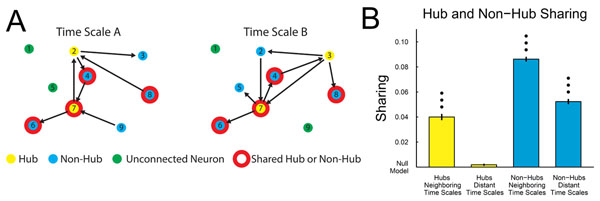
**(A)** Neurons were classified as hubs, non-hubs, or unconnected neurons at all time scales (10 discrete time scales ranging logarithmically from 0.05 – 3 ms to 0.75 – 3 s). **(B)** Hub neurons function as hubs at isolated time scales, while non-hubs operate at many time scales. Null model: chance sharing with matched number of hubs, non-hubs, and unconnected neurons (i.e. independent networks instead of multiplex networks). Significance of sharing values was assessed with a multiple comparison corrected Mann-Whitney test (1, 2, and 3 dots: p < 0.05, 0.01, and 0.001).

Recent studies have emphasized the importance of multiplex networks – interdependent networks with shared nodes and different types of connections – in systems primarily outside of neuroscience [1,2]. Though the multiplex properties of networks are frequently not considered, most networks are actually multiplex networks and the multiplex specific features of networks can greatly affect network behavior (e.g. fault tolerance). Thus, the study of networks of neurons could potentially be greatly enhanced using a multiplex perspective. Given the wide range of temporally dependent rhythms and phenomena present in neural systems, we chose to examine multiplex networks of individual neurons with time scale dependent connections. To study these networks, we used transfer entropy (TE) [3] – an information theoretic quantity that can be used to measure linear and nonlinear interactions – to systematically measure the connectivity between individual neurons at different time scales in cortical and hippocampal slice cultures. We recorded the spiking activity of thousands of neurons across 60 tissue samples using a state-of-the-art 512-electrode array with 60 μm inter-electrode spacing and 50 μs temporal resolution [4]. To the best of our knowledge, this preparation and recording method represents a superior combination of number of recorded neurons and temporal and spatial recording resolutions to any currently available *in vivo* system. We found that highly connected neurons (“hubs”) were localized to certain time scales (Figure 1), which, we hypothesize, increases the fault tolerance of the network. Conversely, a large proportion of non-hub neurons were not localized to certain time scales (Figure 1). In addition, we found that long time scale networks were significantly less modular and more disassortative than short time scale networks in both tissue types. To the best of our knowledge, this analysis represents the first systematic study of temporally dependent multiplex networks among neurons. Furthermore, this analysis demonstrates the ability of transfer entropy to detect effective connectivity at time scales ranging from sub-millisecond to seconds in neural systems. Due to transfer entropy’s general applicability, the methods developed in this work could be adapted to many other types of systems in neuroscience and other disciplines.
